# Age-Related Differences in Cortical and Subcortical Activities during Observation and Motor Imagery of Dynamic Postural Tasks: An fMRI Study

**DOI:** 10.1155/2018/1598178

**Published:** 2018-03-11

**Authors:** A. Mouthon, J. Ruffieux, M. Mouthon, H.-M. Hoogewoud, J.-M. Annoni, W. Taube

**Affiliations:** ^1^Neurology Unit, Medicine Section, Department of Neuroscience and Movement Science, Faculty of Science and Medicine, University of Fribourg, Fribourg, Switzerland; ^2^Department of Radiology, Cantonal Hospital of Fribourg, Fribourg, Switzerland

## Abstract

Age-related changes in brain activation other than in the primary motor cortex are not well known with respect to dynamic balance control. Therefore, the current study aimed to explore age-related differences in the control of static and dynamic postural tasks using fMRI during mental simulation of balance tasks. For this purpose, 16 elderly (72 ± 5 years) and 16 young adults (27 ± 5 years) were asked to mentally simulate a static and a dynamic balance task by motor imagery (MI), action observation (AO), or the combination of AO and MI (AO + MI). Age-related differences were detected in the form of larger brain activations in elderly compared to young participants, especially in the challenging dynamic task when applying AO + MI. Interestingly, when MI (no visual input) was contrasted to AO (visual input), elderly participants revealed deactivation of subcortical areas. The finding that the elderly demonstrated overactivation in mostly cortical areas in challenging postural conditions with visual input (AO + MI and AO) but deactivation in subcortical areas during MI (no vision) may indicate that elderly individuals allocate more cortical resources to the internal representation of dynamic postural tasks. Furthermore, it might be assumed that they depend more strongly on visual input to activate subcortical internal representations.

## 1. Introduction

Aging is associated with deterioration of postural control [1]. This is indicated by an increased variation of the center of pressure, an increase of postural sway, and a higher risk of falls in the elderly [2]. This deterioration has been related to structural and functional changes in the central nervous system [3].

When considering postural control in general, early studies believed that mainly spinal and brainstem structures were involved in postural control [4, 5], although more recent studies showed the crucial importance of cortical and subcortical brain regions [6, 7]. For instance, studies applying transcranial magnetic stimulation (TMS) reported the importance of the primary motor cortex (M1) in the control of dynamic and static postural tasks [7, 8] and showed adaptations in M1 that were correlated with adaptations in balance control [9]. In line with this, structural changes assessed by magnetic resonance imaging (MRI) in the supplementary motor area (SMA) and prefrontal cortex (PFC) were shown to be correlated with changes in postural control, underlining the importance of these cortical areas for postural control [10].

Considering subcortical structures, the cerebellum and basal ganglia were shown to play a fundamental role in balance control. For instance, lesions in those brain regions resulted in severe deterioration of balance [11–14]. Moreover, larger brain activation was found in the cerebellum when a more challenging static postural task was performed [15]. In addition, Goble et al. [16] observed that the level of activation of the basal ganglia during stimulation of the foot muscle spindle was correlated with balance performance indicating that the sensory processing in the basal ganglia is also important for an adequate postural control.

Based on these aforementioned studies, it can be concluded that postural control relies on a brain network involving the PMC, SMA, PFC, M1, brainstem, basal ganglia, and cerebellum. It is important to note that these regions are influenced by aging. For manual motor tasks, numerous functional MRI (fMRI) studies showed greater cortical activity in M1, PMC, and PFC in elderly adults compared to young adults [17–20]. This phenomenon of greater and more diffuse cortical activity in the elderly was observed for both cognitive function and motor function, and it is still debated whether this so-called (cortical) overactivation is related to compensatory processes or dedifferentiation of (i.e., less distinctive) representations [21, 22]. Irrespective of its exact nature, it is reasonable to assume that this age-related overactivation can also be found during the performance of balance tasks. In line with this, changes in the activation of M1 were recently shown by means of TMS during postural tasks. Papegaaij et al. [23] observed a reduction of intracortical inhibition when elderly people were standing on foam compared to standing on a rigid surface while young people displayed unchanged intracortical inhibition. Moreover, a facilitation of motor-evoked potentials during upright stance was observed in the elderly compared to young participants [24, 25], suggesting that aging is accompanied by disinhibition of M1.

In a first step, the current study therefore aimed to clarify whether different ways of mentally simulating postural tasks (action observation = AO, motor imagery = MI, and combination of AO and MI = AO + MI) could reproduce the phenomenon of cortical overactivation known from studies investigating the actual execution of postural tasks. So far, only MI of gait and upright stance was compared between young and elderly subjects using fMRI.

Compared to young adults, elderly participants displayed increased activity in the multisensory vestibular cortices, motion-sensitive visual cortices (MT/V5), and somatosensory cortices (right postcentral gyrus) during motor imagery (MI) of upright stance [26]. The authors supposed that the observed overactivation in the elderly was the result of a reduced reciprocal inhibitory sensory interaction, which may be a compensatory mechanism and may reflect a more conscious postural control. Similarly, an age-related overactivation in the right supplementary motor area (SMA, BA6), the right orbitofrontal cortex (BA11), and the left dorsolateral frontal cortex (BA10) was observed during MI of gait [27].

Taken together, TMS and fMRI studies convincingly demonstrated age-related cortical overactivation during motor tasks in general and postural tasks in particular. However, little is known about differences in subcortical brain regions between young and elderly adults.

Functional MRI studies of the upper extremity reported divergent results when comparing subcortical brain activity in young and elderly participants. Some described reduced activity in the cerebellum or the basal ganglia [28–30] while others observed higher activation levels in subcortical regions in the elderly [19, 20]. With respect to balance control, there is no study to date that compared (subcortical) brain activation levels in young and elderly subjects. This seems important as challenging postural tasks are differently organized than easy postural tasks [31] and age-related differences become more pronounced in complex (e.g., more dynamic) postural tasks [31, 32]. Thus, to better understand postural control and to better tailor nonphysical balance training interventions, it is important to gain a better understanding of age-related changes in the neural processing while mentally simulating postural tasks. In particular, there is a need to clarify the activation of subcortical centers in the aging brain, which is considered to be essential for a more automatic movement execution (e.g., [33]).

Therefore, the main aim of the current study was to explore age-related differences in the internal representation of dynamic postural tasks by means of fMRI in order to detect not only cortical but also subcortical changes. For this purpose, young and older participants were asked to mentally simulate balance tasks by either MI, AO, or the combination of the two (AO + MI) while lying in the scanner. Two postural tasks were simulated: quiet upright stance (static) and the compensation of a mediolateral perturbation (dynamic). As the dynamics of actual balance tasks limit brain accessibility, mental simulation was chosen, which is certainly not a perfect effigy of real task execution but, nevertheless, demonstrates important parallels. It was shown, for instance, that physical task performance and mental simulation activate similar brain areas in a similar manner [34–37]. Based on the activation of overlapping brain areas, Jeannerod [36] postulated the well-accepted theory that “the motor system is part of a simulation network that is activated under a variety of conditions in relation to action, either self-intended or observed from other individuals” (p.103). It is believed that the positive training/learning effects of mental simulation on physical task performance are explained by the activation of this common neural network. With respect to postural control, mental simulation of balance tasks has recently been shown to be effective in improving postural control [38] and to substantially activate motor centers responsible for postural control [39]. It therefore seems reasonable to assume that the activation patterns seen during mental simulation are, indeed, neural representations of postural tasks with a high functional relevance. Thus, the comparison of brain activation patterns in young and elderly adults seems a promising way to assess differences in cortical and subcortical representations of challenging dynamic postural tasks. Based on the results of dual-task studies indicating that processing of motor control shifts from automatic (more subcortical) processing to more consciously controlled (cortical) processing [33, 40], we hypothesized to find greater activation in cortical and lower activation in subcortical areas in old compared to young adults during mental simulation of postural tasks.

Moreover, this design allowed us to investigate age-related differences in the role of vision (or visual guidance) during mental simulation in order to activate internal representations of balance tasks. It is known that elderly people depend more strongly on visual information for postural control than do young adults [41–43] and this dependency was argued to be mainly due to a deterioration of executive functions [44]. In addition, with respect to mental simulation techniques, the combination of AO and MI, the so-called AO + MI, has repeatedly been demonstrated to be more effective than AO or MI alone (for reviews, see [45, 46]). Thus, we speculated that AO + MI might be especially beneficial in the elderly to activate not only cortical but also subcortical brain areas that are important for balancing due to their stronger dependency on visual input with age.

To summarize, the current study aimed to investigate age-related differences in the internal representation of dynamic postural control. We assumed to observe (1) greater activation in cortical and deactivation in subcortical areas as well as (2) larger dependency on visual input to activate internal representations in elderly compared to young people during mental simulation of postural tasks.

## 2. Methods

### 2.1. Participants

Sixteen healthy elderly adults (seven females) aged between 65 and 80 years (mean ± SD = 72 ± 4.6) participated in this study. Their results were compared to the results of a group of sixteen healthy young adults (six females) aged between 20 and 37 years (27 ± 4.8) from a previous study [39]. All participants were free from neurological and orthopedic disorders. They had normal or corrected-to-normal vision. Participants were briefed on the experiments and provided written informed consent for the experimental procedure before testing. The study was approved by the local ethics committee and was in accordance with the Declaration of Helsinki.

### 2.2. Experimental Procedure

**Figure 1:**
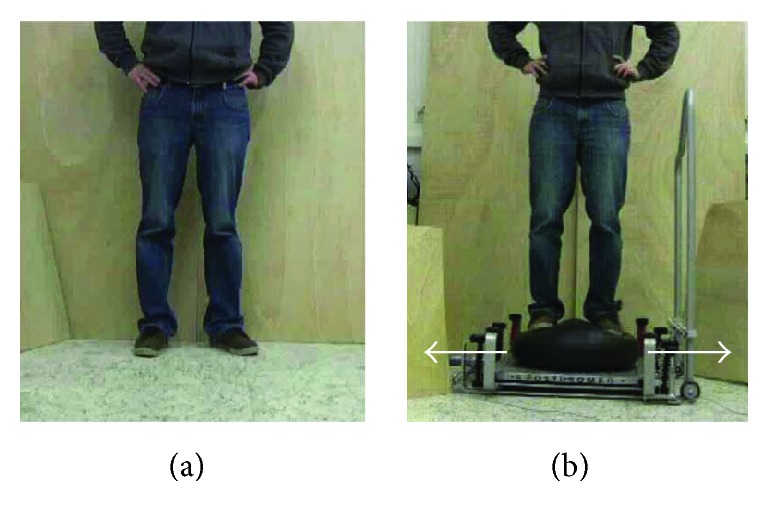
Balance tasks displayed during the experiment. (a) For the static balance task, a person was displayed standing on stable ground. (b) For the dynamic balance task, a person was shown compensating for a mediolateral perturbation while standing on a free-swinging platform (from [47]).

**Figure 2:**
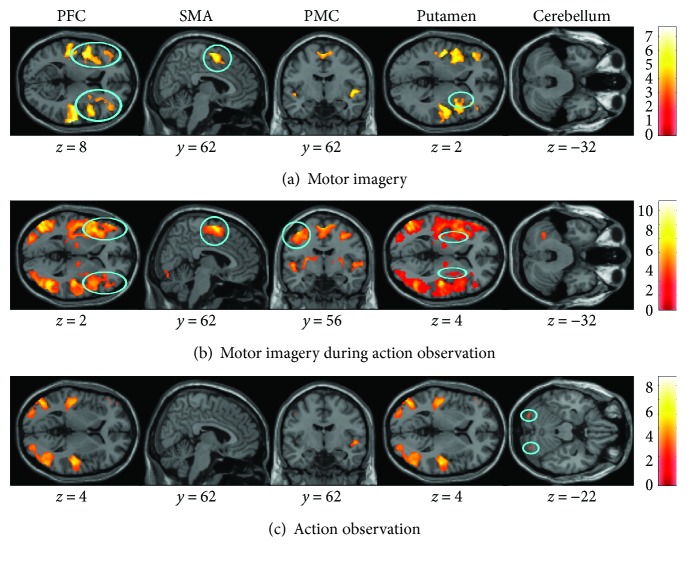
Common brain activity in young and elderly adults detected by a conjunction analysis. The three mental simulation conditions were contrasted with the baseline (mental simulation > baseline). The figure presents shared activities for the dynamic balance task during (a) motor imagery (MI), (b) motor imagery during action observation (AO + MI), and (c) action observation (AO). Colored circles underline significant common brain activity. Whole brain results are presented with *p* < 0.001 at the voxel level, extended by a *p* < 0.05 FWE corrected at the cluster level. Colored bars display the significance level of the contrast. Spatial coordinates (*x*, *y*, and *z*) are provided in MNI space.

The same protocol as in our previous study in young adults [39] was applied to the group of elderly adults. They were instructed to observe or mentally simulate two balance tasks in three different conditions while lying in the scanner. The three mental simulation conditions were (1) AO, (2) MI, and (3) the combination of the two (AO + MI). In the AO condition, the instruction was to merely watch the videos showing a person performing balance tasks. In the MI condition, the participants were asked to imagine themselves performing the respective task with their eyes closed. For the AO + MI condition, the participants were asked to combine the two by watching the video while imagining performing the task themselves at the same time. The MI was performed in a first-person perspective. Two different balance tasks were used in all three mental simulation conditions: (1) standing still on stable ground (Figure 1(a)) and (2) compensating a mediolateral perturbation while standing on a free-swinging platform (Figure 1(b)).

The three mental simulation conditions were tested in separate runs. In the scanner, written and verbal information was provided about which mental simulation condition and which balance task were about to be performed. Each experimental condition (mental simulation) consisted of eight segments (four times each balance task) presented in a random order. Each segment was composed of a 2 s video repeated 10 times, which resulted in video sequences of 20 s, followed by a rest period of 21 s, where a white cross was shown on a black screen. In the video showing the dynamic balance task, each of these 2 s iterations corresponded to one perturbation. In order to notify participants about the start of a new iteration, the start of each video was signaled by a tone. This was particularly important for the MI condition where participants had their eyes closed. All participants were carefully introduced to the tasks and familiarized with the videos by the experimenter before they were placed in the scanner. It was underlined that it was essential that they performed all the tasks only mentally, without any actual movements. The elderly's ability to imagine movements was assessed by a standardized questionnaire (short version of the Kinesthetic and Visual Imagery Questionnaire (KVIQ-10); [48]). In all elderly participants, the average rating of the clarity of the image and the intensity of the sensation was at least three (moderately clear image/moderately intense sensation) on a five-point scale.

### 2.3. Material

Visual stimuli were displayed on an LCD screen (32^″^ LCD Monitor, NordicNeuroLab, Bergen, Norway) with E-Prime 2.0 software (Psychology Software Tools Inc., http://www.pstnet.com, PA, USA) at a refresh rate of 60 Hz. Participants looked at the screen through a mirror system. Auditory information was transmitted through MRI-compatible headphones (Starter f mkII+ MRI Audio System, MR confon, Magdeburg, Germany).

### 2.4. Image Acquisition

Participants were in a supine position in the scanner, and cushions were used to reduce head motion. Data were acquired with a 3T MRI scanner (Discovery MR750, GE Healthcare, Waukesha, Wisconsin, USA) at the Cantonal Hospital of Fribourg, Switzerland (http://www.h-fr.ch). A 32-channel standard head coil was employed for acquisition. High-resolution T1-weighted anatomical scans were collected in the coronal plane (FSPGR BRAVO sequence; voxel size = 0.86 × 0.86 × 1 mm, number of slices = 280, repetition time (TR) = 7300 ms, echo time (TE) = 2.8 ms, and flip angle = 9°; parallel imaging with an acceleration factor of 1.5). Functional T2^∗^-weighted images were recorded with a Gradient Echo-Echo Planar Imaging sequence. The blood oxygenation level-dependent (BOLD) contrast was used as an index of local increases in brain activity. For each experimental session, 150 dynamic volumes with axial acquisitions were collected over the whole brain (voxel size = 1.875 × 1.875 × 3 mm, matrix size = 128 × 128, and number of slices = 40; interleaved acquisition from the bottom to the top of the head, interslice spacing = 0.3 mm, TR = 2500 ms, TE = 30 ms, and flip angle = 85°; parallel imaging with an acceleration factor of 2). To secure steady-state tissue magnetization, the first 7.5 s of each functional run was defined as dummy scans.

### 2.5. Data Processing and Analysis

MRI data were analyzed with the Statistical Parametric Mapping SPM8 software (http://www.fil.ion.ucl.ac.uk/spm) running on MATLAB 2012b (The MathWorks Inc., http://www.mathworks.com, MA, USA). Functional volumes were preprocessed using standard methods implemented in SPM8: spatial realignment, coregistration with anatomical scan, normalization on MNI space (2 × 2 × 2 mm), and smoothing with an isotropic 6 mm full width at half maximum (FWHM) Gaussian kernel. Details have been described previously [39]. The preprocessed images were then subjected to a fixed effect analysis (first-level analysis) based on a general linear model to each voxel [49, 50] for each participant (block design) using an autoregressive [AR(1)] function to account for temporal correlations between voxels across the whole brain.

In a first step, a two-way random effect full factorial model (ANOVA 3 × 2) with the within-subject factor mental simulation condition (AO versus MI versus AO + MI) and balance task (static versus dynamic) was used to estimate brain activity in the elderly group. The pattern of brain activation in each experimental condition (the combination of mental simulation condition and postural task) was studied at the whole brain level by calculating simple effects (contrasts between task and baseline activities, *p* < 0.05 FWE corrected at the voxel level) and direct comparisons between conditions. Brain activation patterns in the young adults have been presented previously [39]. Commonalities as well as differences in brain activation patterns between groups were evaluated by comparing elderly with young people in a second-level analysis. For this purpose, a full factorial model with the age group as a between-subject factor was performed. On the full factorial model, a conjunction analysis [51] was used to determine the common activation of the two groups at the whole brain level (*p* < 0.001 at the voxel level, extended by a *p* < 0.05 FWE corrected at the cluster level and with an extended cluster threshold of 5 contiguous voxels). The FWE-corrected *p* values of the significant clusters are presented in Results.

For the evaluation of age-related differences between groups, a region of interest (ROI) analysis was performed on the full factorial model (*p* < 0.05 FWE at the voxel level and with an extended cluster threshold of 5 contiguous voxels). The FWE-corrected *p* values of the significant voxels are displayed in Results.

Regions analyzed were based on sensorimotor regions that were previously shown to be activated during execution and mental simulation of balance and gait tasks in the literature [14, 15, 27, 52–55]: SMA, PMC, M1, cerebellum, PFC, and putamen.

The locations of ROI were defined with the anatomy toolbox [56], and the locations of the putamen and PFC were based on the automated anatomical labeling (AAL) atlas [57]. To assess the effect of task difficulty on age-related differences in cerebral activity, the interaction between balance task (dynamic versus static balance task) and age group was analyzed for each mental simulation condition (AO + MI, AO, and MI). Finally, to investigate the effect of aging on the type of mental simulation and the impact of visual guidance, the interaction between mental simulation condition (AO + MI versus AO versus MI) and age group was analyzed for both balance tasks.

## 3. Results

In the following, brain activation patterns in elderly adults are described first. Afterwards, common activity (revealed by the conjunction analysis) in young and elderly adults is displayed before describing differences in brain activation between the two groups. The results for the young participants have recently been presented elsewhere [39].

### 3.1. Brain Activity Pattern in Elderly Adults

#### 3.1.1. Simple Effects

For elderly adults, activity in brain regions important for postural control was detected in all mental simulation conditions for the dynamic postural task. During MI of the dynamic balance task, activities in the bilateral SMA (*p* < 0.001), right PMC (*p* = 0.006), bilateral PFC (*p* < 0.001), and left putamen (*p* < 0.001) were observed. The AO + MI condition of the dynamic task involved the bilateral SMA (*p* < 0.001), bilateral M1 (*p* < 0.001), bilateral putamen (*p* = 0.001), right PMC (*p* < 0.001), bilateral PFC (*p* < 0.001), and left cerebellum (*p* < 0.001, lobules I–IV and lobule VI). Furthermore, during AO of the dynamic task, the right SMA (*p* = 0.03), right PFC (*p* < 0.001), and left cerebellum (*p* = 0.03; lobules VIIa and VIIb) were activated. The static balance task induced activation in the right PMC (*p* = 0.01) and right PFC (*p* < 0.001) during MI and in the right SMA (*p* = 0.02) during AO + MI. Interestingly, no significant activity was detected in brain areas associated with balance control during AO of the static balance task. We also found activity in areas processing visual and auditory information (results not illustrated).

#### 3.1.2. Complexity of the Balance Task

In order to investigate whether the complexity of the balance task had an influence on the activation of brain centers in elderly adults, the dynamic balance task was compared to the static task. During AO + MI, this comparison displayed stronger activation in the bilateral SMA (*p* < 0.001), bilateral PMC (*p* < 0.001), bilateral PFC (*p* = 0.001), and left cerebellum (*p* < 0.001; lobules I–IV and V) for the dynamic task (results are presented in Table 1 of Supplementary Materials which provide the table of brain activities observed in the elderly when the dynamic balance is compared with the static task during action observation combined with motor imagery (AO+MI)). No significant differences between the dynamic and the static tasks were detected for the MI and AO conditions.

#### 3.1.3. Effect of Mental Simulation Condition

There were no significant differences in brain activation in areas important for balance control between mental simulation conditions for the dynamic task when comparing AO + MI with MI and MI with AO. However, when AO + MI was compared with AO, higher activations were seen in the left putamen (*p* < 0.001), bilateral SMA (*p* < 0.001), and bilateral PMC (*p* < 0.001).

Motor imagery during action observation (AO + MI) of the dynamic task did not equal the sum of the AO and MI conditions. Indeed, the elderly presented significantly larger activation during AO + MI than the sum of brain activity during independent MI and independent AO in the bilateral M1 (*p* < 0.001), bilateral SMA (*p* < 0.001), left PMC (*p* < 0.001), bilateral cerebellum (*p* < 0.005), and left putamen (*p* < 0.001).

### 3.2. Common Activity in Young and Elderly Adults

In order to identify brain regions that were activated in both age groups, a conjunction analysis was conducted for each mental simulation condition (Figure 2). Common activation was observed in the bilateral SMA (dynamic: *p* < 0.001; static: *p* = 0.05) and bilateral PFC (dynamic: *p* < 0.001; static: *p* < 0.001) for MI of both balance tasks and in the right putamen (*p* < 0.001) only for the dynamic task. During AO + MI of both balance tasks, common activation was observed in the bilateral SMA (dynamic: *p* < 0.001; static *p* = 0.009), bilateral PMC (dynamic: *p* < 0.001; static: *p* = 0.01), the left putamen (dynamic: *p* < 0.001; static: *p* < 0.001), and PFC (dynamic: *p* < 0.001; static: *p* < 0.001). Common activity was further seen in the bilateral cerebellum (lobule VIIa Crus I, *p* < 0.001) during AO of the dynamic balance task, but no common cerebral activation was found for the static task. Not surprisingly, there was also common activity in areas processing visual and auditory information (results not illustrated).

### 3.3. Age-Related Differences in Brain Activity

#### 3.3.1. Simple Effects

**Figure 3:**
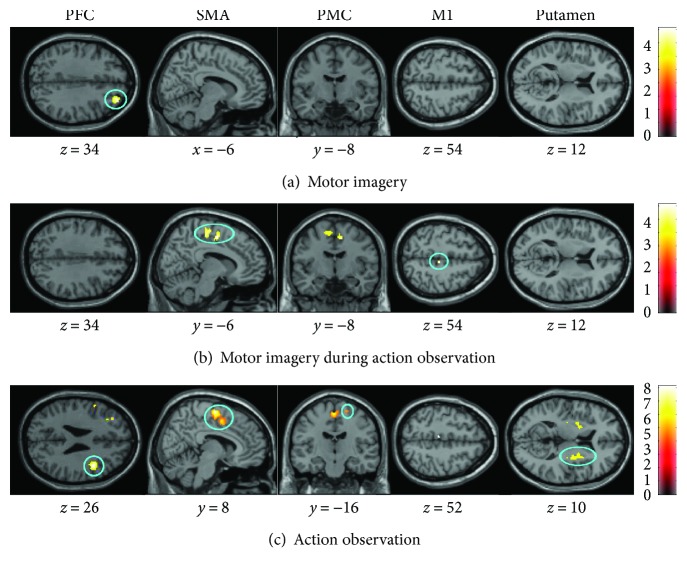
Age-related differences in brain activity. The three mental simulation conditions were contrasted between age group by means of an ROI analysis. Presented are significantly different activities for the dynamic balance task during (a) motor imagery (MI), (b) motor imagery during action observation (AO + MI), and (c) action observation (AO). Colored circles highlight significantly greater brain activity in elderly adults. Activations are presented with *p* < 0.001 at the voxel level, extended by a *p* < 0.05 FWE corrected at the cluster level. Colored bars indicate the significance level of the contrasts. Spatial coordinates (*x*, *y*, and *z*) are provided in MNI space.

In order to detect differences in brain activity between elderly and young adults, comparisons of the simple effects (conditions compared to the baseline, Figure 3) were performed by means of an ROI analysis. The AO + MI of the dynamic task condition induced stronger activity in SMA (*p* = 0.01) and M1 (*p* = 0.03) in elderly compared to young people. No significant difference between groups was found for AO + MI of the static task. AO of the dynamic task induced larger activity in SMA (*p* < 0.001), PMC (*p* = 0.05), PFC (*p* = 0.03), and putamen (*p* = 0.01) in elderly compared to young participants. AO of the static task revealed increased brain activity only in the SMA (*p* = 0.02). During MI, larger brain activations were observed in older individuals compared to young adults in the PFC (*p* = 0.04) for the dynamic task and in the putamen (*p* = 0.01) for the static task. The younger adults did not present significantly higher brain activity than the elderly individuals in any mental simulation condition.

#### 3.3.2. Complexity of the Balance Task

To evaluate age-related differences in the effects of balance task type, task effects in the two groups were compared. During AO + MI, an ROI analysis revealed that the effect of task (dynamic task > static task) was greater in old individuals compared to the young adults in the SMA (*p* = 0.04) and PFC (*p* = 0.001). No significant age-related differences in task-specific effects were found for MI and AO.

#### 3.3.3. Effect of Mental Simulation Condition

**Figure 4:**
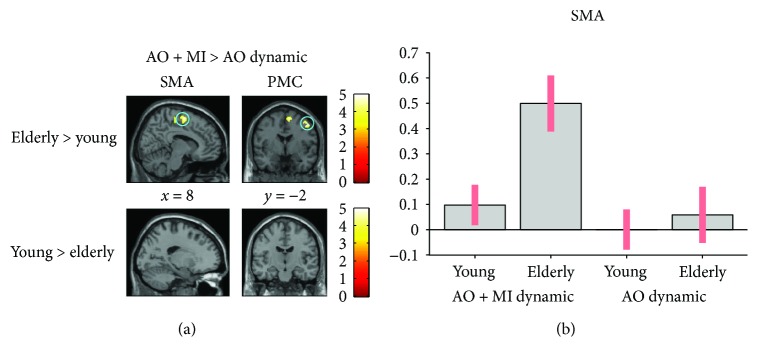
Age-related differences in brain activity when motor imagery during action observation (AO + MI) is contrasted with action observation (AO), revealed by an ROI analysis. (a) presents differences in activity in the SMA (*p* = 0.02) and PMC (*p* = 0.02) between groups. (b) shows the activation level of a representative voxel (8, −24, and 58) of the SMA. Colored circles highlight significantly stronger brain activity. Activations are presented with *p* < 0.001 at the voxel level, extended by a *p* < 0.05 FWE corrected at the cluster level. Colored bars indicate the significance level of the contrasts. Spatial coordinates (*x*, *y*, and *z*) are provided in MNI space.

*(1) AO + MI versus AO*. An ROI analysis for this contrast indicated larger activation of the bilateral SMA (*p* = 0.02) and the right PMC (*p* = 0.02) in the elderly group for the dynamic task (see Figure 4). In contrast, young subjects did not show higher activation than elderly participants in any brain area for the dynamic task. For the static task, the young presented stronger activation in the cerebellum (*p* = 0.01; lobule VIIa Crus I, lobule VIIIb, and lobule VI).

**Figure 5:**
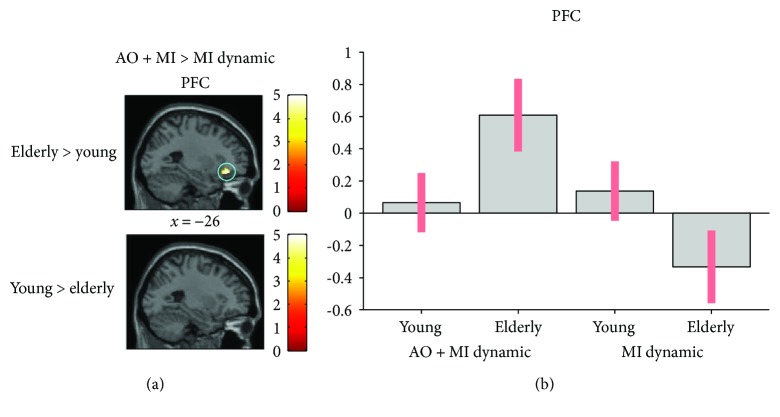
Age-related differences in brain activity when motor imagery during action observation (AO + MI) is contrasted with motor imagery alone (MI), revealed by an ROI analysis. (a) shows higher brain activity for the dynamic task in the PFC (*p* = 0.05) in older adults when compared to young adults. (b) shows the activation level of a representative voxel (−28, 34, and −14) of the PFC depending on the group and the mental simulation condition (AO + MI or MI). Colored circles highlight significantly stronger brain activity in elderly adults. Activations are displayed with *p* < 0.001 at the voxel level, extended by a *p* < 0.05 FWE corrected at the cluster level. Colored bars indicate the significance level of the contrasts. Spatial coordinates (*x*, *y*, and *z*) are provided in MNI space.

*(2) AO + MI versus MI*. The comparison of AO + MI versus MI of the dynamic task revealed greater activation in the PFC (*p* = 0.05) in elderly individuals (see Figure 5).

**Figure 6:**
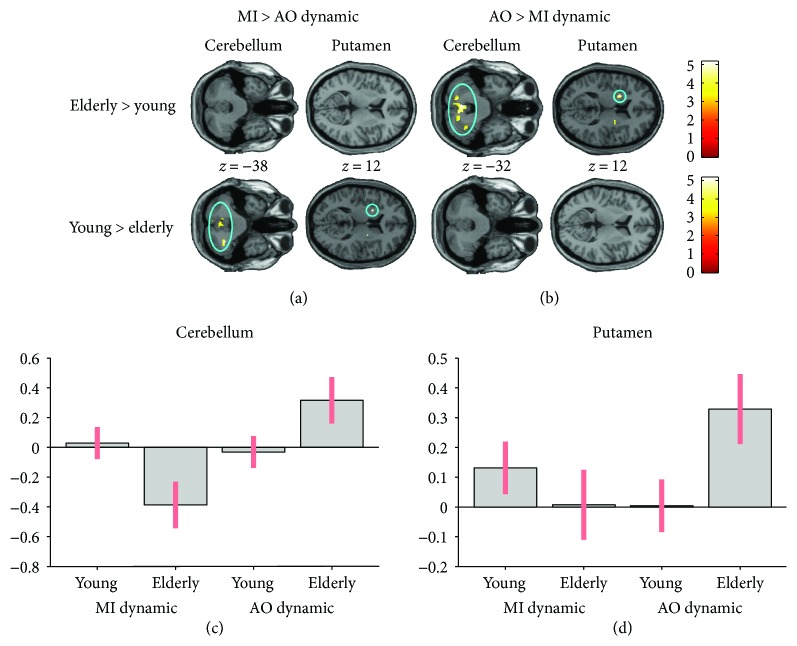
Age-related differences in the interactions between brain activity during motor imagery (MI) and action observation (AO), revealed by an ROI analysis. (a) shows higher brain activity in the cerebellum (*p* = 0.02) and putamen (*p* = 0.05) in young adults compared to older adults, when a condition with no visual support (MI) is contrasted with one with visual support (AO) for the dynamic task. Inversely, (b) presented greater cerebral activity in the cerebellum (*p* = 0.02) and putamen (*p* = 0.008) in elderly adults compared to young adults, when a condition with visual support (AO) is contrasted with one with no visual support (MI). (c) and (d) represent the activation of the cerebellum and the putamen depending on the group and the mental simulation condition. The plots for the cerebellum and the putamen are based on voxels (−2, −60, and −34 and 30, −18, and 2, resp.). Colored circles highlight significantly stronger brain activity in young adults. Activations are displayed with *p* < 0.001 at the voxel level, extended by a *p* < 0.05 FWE corrected at the cluster level. Colored bars indicate the significance level of the contrasts. Spatial coordinates (*x*, *y*, and *z*) are provided in MNI space.

*(3) MI versus AO*. When comparing the interaction between MI and AO (MI > AO) in the two age groups, an ROI analysis revealed that in the cerebellum (*p* = 0.02; lobule VIIa Crus I) and the putamen (*p* = 0.05), young adults had greater cerebral activity than the elderly for the dynamic task (see Figure 6). There was no difference between groups in the static task.

## 4. Discussion

This first study about different ways to mentally simulate dynamic postural tasks in old and young participants revealed a considerable amount of common brain activity between elderly and young adults. However, there were also marked differences in the activation patterns of elderly participants evidenced by greater cortical activation in PFC, SMA, PMC, M1, and putamen. Moreover, when MI (no visual input) was contrasted to AO (visual input), the elderly, relative to young participants, demonstrated deactivation of subcortical areas such as the cerebellum and the putamen in the condition with no visual input. Thus, elderly individuals appear to rely more on visual guidance to activate subcortical representations of balance tasks and it might therefore be even more important to combine AO with MI (AO + MI) in elderly than in young subjects.

### 4.1. Similarities in Cerebral Activity between Age Groups

The current results demonstrate that both young and elderly adults activated brain regions involved in postural control when they observed and/or imagined a postural task. In line with recent observations for nonpostural [26, 27, 58] and our previously reported results for postural tasks in young participants [39], elderly participants demonstrated greatest activity during AO + MI, followed by MI and AO. Moreover, brain activities observed in the M1, SMA, PMC, cerebellum, and putamen during AO + MI were not simply the addition of activity of independent AO and independent MI but were significantly larger than the sum of those two conditions. This finding supports previous studies reporting the phenomenon of supra-additive effects of AO + MI compared to AO or MI alone (e.g., Sakamoto et al. and Taube et al. [39]).

It was recently speculated that the combination of AO + MI may enable subjects to gain better physiological sensations and kinesthetic experiences of the imagined movement [45]. Importantly, the present study demonstrates that this supra-additive effect can also be seen in elderly subjects and therefore proposes that AO + MI is a very promising approach to activate internal movement representations of postural tasks in the elderly.

The importance of combining AO + MI in the elderly was also apparent, when combining the two different balance tasks. Only with AO + MI, the more demanding dynamic balance task induced significant greater activation in brain regions important for postural control than the simple balance task. This result is also in line with previous findings in young adults showing larger effects for the more demanding postural task [39]. Therefore, it is not surprising that when directly assessing common activity of brain centers in elderly and young people by means of a conjunction analysis, considerable overlap could be observed. However, despite these similarities there were also distinct differences between young and elderly participants.

### 4.2. Differences in Cerebral Activity between Age Groups

Compared to young adults, elderly participants displayed greater activity in the SMA, M1, PMC, and putamen during mental simulation of the dynamic balance tasks. For the upper extremity, most studies associated such an age-specific overactivation with better motor performance compared to age-matched subjects that did not show overactivation/disinhibition [18–20]. Therefore, it is generally believed that increased cortical activity and reduced cortical inhibition serve as compensatory mechanisms for structural degeneration (“compensation hypothesis”; for review, see [3]). Alternatively, it was suggested that elderly adults might present a more nonselective recruitment of brain regions (“dedifferentiation hypothesis”; [22]). However, this second hypothesis seems less likely, as elderly adults showed a very similar task-dependent pattern of activity as young adults (conjunction analysis) in the present study. In line with this, greater activations were seen in the cerebellum and SMA, when the dynamic task was contrasted with the static task during the AO + MI condition in both populations. However, comparison of elderly with young adults for this contrast revealed stronger activation of the SMA and PMC in the elderly. These results indicate that age-related changes were more prominent in the more complex dynamic postural task than in the static standing task. Furthermore, as these differences were only significant in the AO + MI condition, this finding supports the assumption of greater efficacy of AO + MI compared to AO or MI alone (for review, see [45]). From a motor control point of view, it can be argued that elderly people relied more strongly on cortical areas to active internal representations of the dynamic postural task. Alternatively, the age-related difference observed in this study could also indicate the decline of cognitive function in the elderly. However, we can only speculate regarding the latter, as the participants' cognitive abilities were not assessed, which is one limitation of this study. Nonetheless, as all our elderly participants were able to adequately perform MI, we do not think that cognitive abilities were substantially different between groups. This is in line with previous studies on motor imagery reporting that the vividness is well preserved in the elderly [59, 60]. In addition, elderly individuals were shown to present similar temporal congruence between MI and movement execution to young adults [61–64]. Therefore, we reckon that the age-related changes detected in this study more likely indicate a decline of the mental ability to simulate challenging postural tasks than a general deterioration of the ability to perform mental simulation. This is further supported by the fact that there were no age-related differences when comparing the static postural task.

The age-related overactivation in cortical brain areas during mental simulation of the dynamic balance task confirms data from neurophysiological measurements during actual postural task execution. By means of TMS, several studies demonstrated increased corticospinal excitability in elderly compared to young people when performing balance tasks [25, 65]. In addition, intracortical inhibition was shown to be reduced in elderly compared to young participants in challenging postural conditions [23]. Thus, age-related overactivation of cortical areas seems to be apparent during both actual task execution and mental simulation of dynamic postural tasks. Unfortunately, measurements during balance activities are restricted to motor cortical areas accessible with TMS. Therefore, little is known about age-specific activity of subcortical brain regions during postural task execution. Furthermore, there exist no studies comparing young and elderly adults during mental simulation of dynamic postural tasks.

Allali et al. [27] investigated brain activation while subjects were asked to imagine walking over even ground or over cobblestones. In general, elderly adults demonstrated larger activation than did young adults in the left middle frontal gyrus (BA10), right SMA, and right superior orbitofrontal cortex (BA11). Furthermore, compared to young subjects, elderly participants displayed stronger activation in the left hippocampus when switching from walking over even ground to the more challenging task of walking over cobblestones [27]. However, although balance certainly plays a considerable role during walking, it may, nevertheless, not be entirely comparable to other postural tasks such as upright stance. For instance, when evaluating age-dependent alterations during imagined walking, running, and standing, Zwergal et al. [26] demonstrated the greatest multisensory activation during standing followed by walking and finally running in the elderly. The authors argued that gait relies strongly on subcortical locomotor centers that are evolutionarily old structures and, therefore, probably less susceptible to atrophy or dysfunction in advanced age. In contrast, unperturbed stance [8] and perturbed stance [7] were shown to rely on motor cortical areas, and cortical plasticity in these regions was correlated with behavioral adaptations after balance training [9, 55, 66]. Thus, the (cortical) control of dynamic upright stance may be more prone to aging. This may also explain the pronounced differences in cortical centers that were detected in the present study. Compared to young adults, elderly participants displayed greater activity in the SMA, M1, and PMC during mental simulation. However, there were also differences in subcortical brain activation patterns. However, these differences seemed to strongly depend on whether or not elderly subjects received “visual guidance” during mental simulation of postural tasks.

### 4.3. Impact of Visual Input for the Mental Simulation of Postural Tasks

In the current study, different types of mental simulation (AO + MI versus AO versus MI) were compared in terms of activating brain centers responsible for postural control in young and elderly subjects. In the elderly, stronger cortical activity was mainly found for the conditions with visual input (AO + MI and AO). In the condition without visual input (MI), elderly participants displayed solely facilitation in the PFC while reduced activity was found in subcortical areas such as the putamen and the cerebellum. Therefore, it seems that, in elderly adults, the activation of subcortical representations responsible for the control of (perturbed) stance strongly depended on visual input. Activation in these areas is considered important for automated (postural) task execution [67]. Based on mainly dual-task findings, the group around Stephan Swinnen stressed that elderly adults exhibit less automatic processing of upright posture, resulting in greater activation of cognitive resources [33]. Furthermore, dual-task studies investigating the influence of vision demonstrated that dual-task costs, that is, the reduction in performance due to the execution of a concurrent secondary task, were especially pronounced in elderly adults when secondary tasks that required substantial visual processing were chosen [42, 44]. Thus, the findings of the current study may provide a first indication that, for elderly people, visual input is important during mental simulation to better activate subcortical brain centers that enable more automatized movement control. When vision is removed (MI condition), elderly participants display a decrease of activation of these subcortical areas while, at the same time, activity in the PFC increases, indicating the greater involvement of cognitive resources.

### 4.4. Functional Considerations and Conclusion

Elderly and young adults demonstrated very similar brain activation patterns when mentally simulating postural tasks. Both age groups increased brain activity in the dynamic postural task and when combining AO with MI (AO + MI). Thus, for both age groups, interventions involving mental simulation should involve dynamic (complex) postural tasks and AO + MI. The comparison of brain activation in young and elderly participants between mental simulation conditions proposes that the combination of AO and MI might be even more important for elderly people. Indeed, in conditions with visual input (AO + MI and AO), the elderly demonstrated greater cortical activity whereas in the condition without visual input (MI), they showed solely facilitation in the PFC but a decrease in activity in subcortical areas such as the putamen and the cerebellum. Our results, therefore, indicate that the activation of internal representations of postural tasks by means of mental simulation in elderly people depends more strongly on visual input than that in young participants. This visual input seems especially important to activate subcortical brain centers, such as the cerebellum and the basal ganglia, which are probably important to enable automatized task execution. Consequently, nonphysical balance training in elderly adults should use visual guidance to promote activity in these brain areas.

## Supplementary Materials

Supplementary Materials Table 1: brain activity observed in the elderly when the dynamic balance is compared with the static task during action observation combined with motor imagery (AO + MI). The table presents all significant brain activations observed in the condition. The spatial location (coordinates *x*, *y*, and *z*) of the voxel with the highest *Z*-score (*Z*-max) inside of each cluster is presented.
