# Supplementary material for: Age-Related Differences in Cortical and Subcortical Activities during Observation and Motor Imagery of Dynamic Postural Tasks: An fMRI Study
Source: Neural Plast. 2018 Mar 11;2018:1598178. doi: 10.1155/2018/1598178 (PMC5872650; doi:10.1155/2018/1598178)
Supplement: Supplementary Materials — Table 1: brain activity observed in the elderly when the dynamic balance is compared with the static task during action observation combined with motor imagery (AO + MI). The table presents all significant brain activations observed in the condition. The spatial location (coordinates x, y, and z) of the voxel with the highest Z-score (Z-max) inside of each cluster is presented. [file 1598178.f1.docx]

Supplementary material

Table 1: Brain activity observed in the elderly when the dynamic balance is compared with the static task during action observation combined with motor imagery (AO+MI). The table presents all significant brain activations observed in the condition. The spatial location (coordinates x, y, and z) of the voxel with the highest Z-score (Z-max) inside of each cluster is presented.

| Anatomic location | *X* | *Y* | | *Z* | Cluster size | | Z-max |
| --- | --- | --- | --- | --- | --- | --- | --- |
|  |  |  |  | |  |  | |
| Left Middle Occipital Gyrus | -52 | -72 | 0 | | 1652 | 65535 | |
|  | -42 | -68 | 4 | |  | 7.49 | |
|  | -56 | -64 | 0 | |  | 6.58 | |
| Right Middle Temporal Gyrus | 52 | -64 | 2 | | 2987 | 65535 | |
| Right Rolandic Operculum | 54 | -30 | 22 | |  | 7.13 | |
| Right Superior Temporal Gyrus | 62 | -32 | 22 | |  | 7.12 | |
| Left SMA | 0 | -10 | 56 | | 19118 | 6.30 | |
|  | 0 | -10 | 56 | |  | 6.30 | |
|  | -8 | 10 | 44 | |  | 5.44 | |
| Right SMA | 8 | -6 | 64 | |  | 6.16 | |
|  | 8 | -6 | 64 | |  | 6.16 | |
|  | 6 | -2 | 52 | |  | 5.94 | |
|  | 8 | -6 | 54 | |  | 5.75 | |
| Right Rolandic Operculum | 52 | 6 | 8 | |  | 6.24 | |
|  | 52 | 6 | 8 | |  | 6.24 | |
| Left Rolandic Operculum | -50 | 0 | 8 | |  | 5.96 | |
| Right Superior Frontal Gyrus | 16 | -18 | 68 | |  | 5.75 | |
| Right Thalamus | 18 | -16 | 10 | |  | 5.49 | |
| Left Thalamus | -12 | -16 | 2 | |  | 5.71 | |
| RightPrecentral Gyrus | 60 | 8 | 18 | |  | 5.70 | |
| Left Insula Lobe | -28 | 22 | -6 | |  | 5.65 | |
| Right Inferior Frontal Gyrus (p. Opercularis) | 42 | 16 | 4 | |  | 5.54 | |
| Left Insula Lobe | -40 | 12 | 4 | |  | 5.50 | |
| Left Paracentral Lobule | -16 | -24 | 68 | |  | 5.47 | |
| Right Middle Cingulate Cortex | 6 | 10 | 44 | |  | 5.45 | |
| Left Cerebellum | -10 | -46 | -18 | | 812 | 5.19 | |
|  | -4 | -48 | -12 | |  | 5.15 | |
|  | -10 | -46 | -18 | |  | 5.19 | |
|  | -4 | -48 | -12 | |  | 5.15 | |
|  | -10 | -68 | -18 | |  | 3.38 | |
| Cerebellar Vermis | 4 | -52 | -22 | |  | 4.73 | |
|  | 4 | -52 | -22 | |  | 4.73 | |
|  | 4 | -50 | -6 | |  | 4.30 | |
|  | 4 | -78 | -14 | |  | 4.21 | |
|  | 0 | -62 | -12 | |  | 4.00 | |
|  | 0 | -62 | -20 | |  | 3.87 | |
| Right Cerebellum | 12 | -44 | -20 | |  | 3.85 | |
|  | 8 | -60 | -20 | |  | 3.22 | |
| Left Calcarine Gyrus | -2 | -86 | -12 | |  | 3.42 | |
